# Connectome and regulatory hubs of CAGE highly active enhancers

**DOI:** 10.1038/s41598-023-32669-3

**Published:** 2023-04-05

**Authors:** Mewen Briend, Anne Rufiange, Louis-Hippolyte Minvielle Moncla, Samuel Mathieu, Yohan Bossé, Patrick Mathieu

**Affiliations:** 1 Genomic Medicine Laboratory, Quebec Heart and Lung Institute, Laval University, Quebec, Canada; 2 Quebec Heart and Lung Institute, Laval University, Quebec, Canada; 3 Department of Molecular Medicine, Laval University, Quebec, Canada; 4 Institut de Cardiologie et de Pneumologie de Québec/Québec Heart and Lung Institute, 2725 Chemin Ste-Foy, Québec, Québec G1V-4G5 Canada

**Keywords:** Genome, Genomics

## Abstract

Evidence indicates that enhancers are transcriptionally active. Herein, we investigated transcriptionally active enhancers by using cap analysis of gene expression (CAGE) combined with epigenetic marks and chromatin interactions. We identified CAGE-tag highly active (CHA) enhancers as distant regulatory elements with CAGE-tag ≥ 90th percentile and overlapping with H3K27ac peaks (4.5% of enhancers). CHA enhancers were conserved between mouse and man and were independent from super-enhancers in predicting cell identity with lower P-values. CHA enhancers had increased open chromatin and a higher recruitment of cell-specific transcription factors as well as molecules involved in 3D genome interactions. HiChIP analysis of enhancer-promoter looping indicated that CHA enhancers had a higher density of anchor loops when compared to regular enhancers. A subset of CHA enhancers and promoters characterized by a high density of chromatin loops and forming hub regulatory units were connected to the promoter of immediate early response genes, genes involved in cancer and encoding for transcription factors. Promoter of genes within hub CHA regulatory units were less likely to be paused. CHA enhancers were enriched in gene variants associated with autoimmune disorders and had looping with causal candidate genes as revealed by Mendelian randomization. Hence, CHA enhancers form a dense hierarchical network of chromatin interactions between regulatory elements and genes involved in cell identity and disorders.

## Introduction

The regulation of gene expression relies on complex interactions between gene promoters and distant *cis*-acting elements often referred to as enhancers^1^. Enhancers, which often reside several kilobases away from their target protein coding genes, are enriched in cell-specific transcription factor motifs^2^. As such, enhancers participate in cell identity and developmental program^3^. The mechanism by which enhancers control the expression of genes involves the looping process, which allows two distant regions on the linear genome to be in close proximity^4^.

Studies have underlined that active enhancers are enriched in the histone mark H3K27ac, which is often used to assess the presence of distant-active regulatory elements^5^. H3K27ac mark, which is deposited by the acetyltransferase EP300^6^, allows the recruitment of transcriptional cofactors such as bromodomain containing protein BRD4^7^. Hence, acetylation of histone is an actively regulated process, which participates to the activity of regulatory elements. Distant-acting elements are associated with the recruitment RNA polymerase II (RNAPII), which produces enhancer-associated RNAs (eRNAs)^8,9^. Based on cap analysis of gene expression (CAGE)^10^, the Functional Annotation of the Mammalian Genome (FANTOM5)^11^ consortium has underlined that transcription is pervasive in the noncoding genome, often bidirectional and associated with the expression of protein coding genes. FANTOM5 has produced an exhaustive list of actively transcribed enhancer regions in different cell lines and primary cells^12,13^.

According to the number of connections, enhancers may exert hierarchical activity and establish enhancer-promoter networks^14,15^. Several processes are likely involved in the control of looping. The loop extrusion model in which CTCF and the cohesin complex interact explains how topologically associated domains (TADs) are established^16,17^. Also, recruitment of transcription factors and RNA polymerase II (RNAPII) are significant features that participate to the establishment of intra-domain interactions between enhancers and promoters^18^. Herein, in H3K27ac defined enhancers we leveraged CAGE analysis to assess: (1) the relationship between enhancer-associated CAGE activity and the chromatin landscape at regulatory elements, (2) the association of enhancer-associated CAGE activity with 3D genome organization, (3) whether looping between enhancer-associated CAGE activity and promoter defines a subset of regulatory units (i.e. loop between a promoter and its enhancers) and (4) whether enhancer-associated CAGE activity is informative for the mapping of disease-associated gene variants to target causal genes. We underlined that a small proportion of enhancers, referred to as CAGE-tag highly active (CHA) enhancers, were associated with the recruitment of cell-specific transcription factors, cell function and a high density of enhancer-promoter contacts. CHA enhancers were also enriched in gene variants associated with autoimmune disorders and had looping with causal candidate genes. A subset of highly 3D connected CHA enhancer-promoter units were associated with transcription elongation at protein coding genes involved in key cellular functions. Our findings establish a distinct role for CHA enhancers in chromatin organization and in enhancer-promoter networks with relevance for cell function and disease.

## Results

### Transcriptionally active enhancers

**Figure 1:**
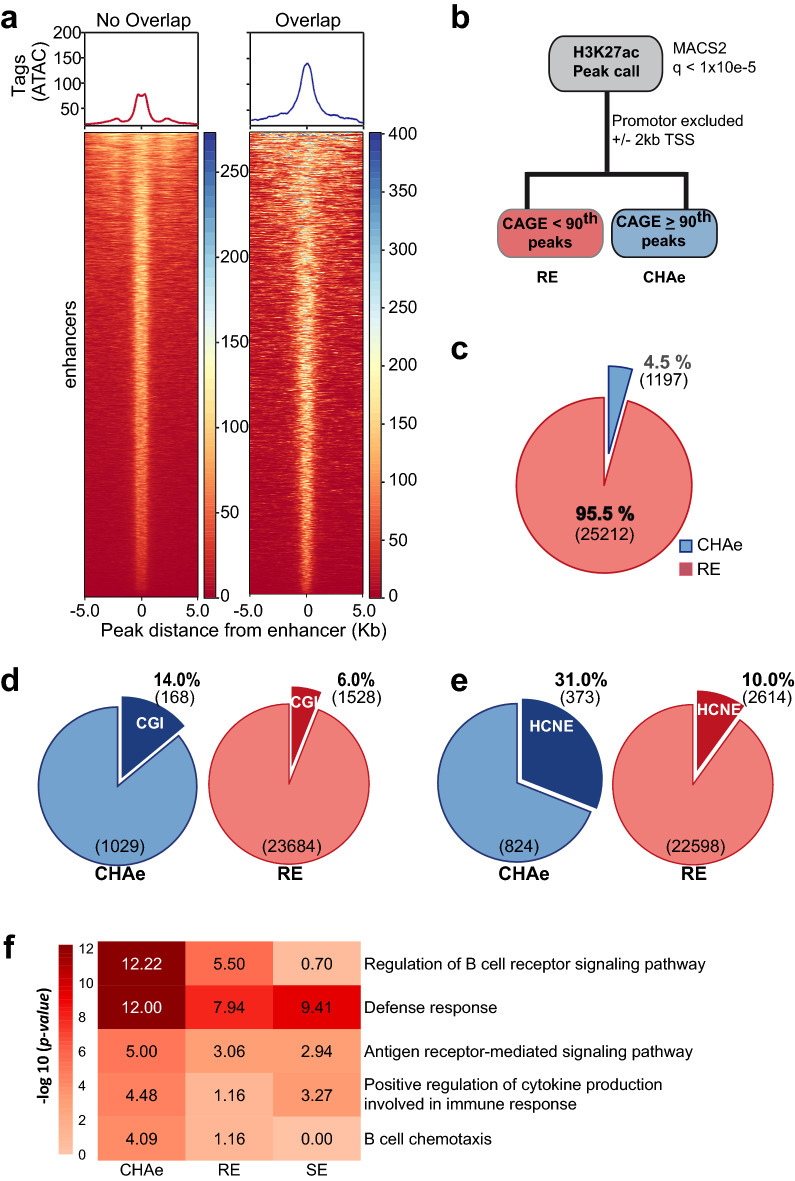
Characterisation of transcriptionally active enhancers. (**a**) Heat map representing ATAC activity (open chromatin) in H3K27ac-defined enhancers with or without overlap with nonzero CAGE-tag regions (deeptools, plotHeatMap). (**b**) Pipeline to identify CHAe and RE. (**c**) Proportions of CHAe and RE. (**d**) Proportions of CHAe and RE overlapping with CGI (P=2.96E-22, Fisher’s exact test). (**e**) Proportions of CHAe and RE overlapping with HCNE between human and mouse (P=1.21E-80, Fisher’s exact test). (**f**) GO enrichment of CHAe, RE and SE. *CHAe* CAGE-tag highly active enhancer, *RE* regular enhancer, *SE* super-enhancer, *CGI* CpG island, *HCNE* human conserved noncoding elements (between man and mouse), *GO* gene ontology.

We first set out to identify distant regulatory elements in blood B cell line (GM12878) by using H3K27ac (data from ENCODE)^19–21^. ChIP-seq defined enhancers were identified from H3K27ac peaks and chromatin active regions overlapping gene promoters were excluded (“Methods”). In total, 26,409 regions were identified as enhancers. We next leveraged CAGE data from the FANTOM5 consortium to assess the overlap of transcriptionally active regions with H3K27ac-defined enhancers. H3K27ac is required for the transcriptional activity of enhancer RNA (eRNA)^22^. Accordingly, in the 26,409 enhancers previously identified, CAGE-tag correlated positively with H3K27ac (spearman correlation = 0.35) (Supplementary Fig. 1). Among the H3K27ac enhancers, those overlapping with active CAGE regions had much higher open chromatin identified by transposase accessible chromatin and high-throughput sequencing (ATAC-seq) assay (Fig. 1a). According to the level of CAGE activity, we found that higher transcription (based on CAGE percentile) at H3K27ac defined enhancers was associated with elevated open chromatin (Supplementary Fig. 2). Considering the previous finding, we defined CAGE-tag highly active (CHA) enhancers as those with CAGE-tag activity ≥ 90th percentile, which overlapped H3K27ac peaks (Fig. 1b). The intersection of CAGE-tag ≥ 90th percentile with H3K27ac peak regions identified 1197 (1197/26409, 4.5% of enhancers) CHA enhancers (Fig. 1c). CHA enhancers were compared to H3K27ac defined enhancers with CAGE-tag activity < 90th percentile including regions without detectable CAGE-tag activity, which were referred to as regular enhancers. We found that 14% of CHA enhancers vs. 6% of regular enhancers overlapped with CpG islands (CGI) (P=2.96E − 22, Fisher’s exact test) (Fig. 1d). Also, a higher proportion of CHA enhancers intersected with highly conserved noncoding elements between human and mouse (31% vs. 10% respectively for CHA and regular enhancers, P=1.21E − 80, Fisher’s exact test) (Fig. 1e). As CHA enhancers were conserved, we hypothesized that these regions may confer cell-specific functions. Annotation of gene ontology (GO) showed an enrichment of lower P-values for cell-specific functions in CHA vs. regular enhancers. As super-enhancers (SEs), which are group of enhancers in close proximity, have been shown to be associated with cell identity^3^, we compared the overlap between CHA enhancers and SEs. By using H3K27ac we identified 615 SEs in GM12878. We found a poor overlap between the CHA enhancers and SEs (Jaccard index=0.03). Assessment of P-values for cell-specific functions in GO showed lower P-values in CHA enhancers vs. SEs (Fig. 1f). Taken together, the present findings suggest that CHA enhancers are largely independent from SEs and are associated with cell identity.

### Chromatin landscape and transcription factors in CHA enhancers

**Figure 2:**
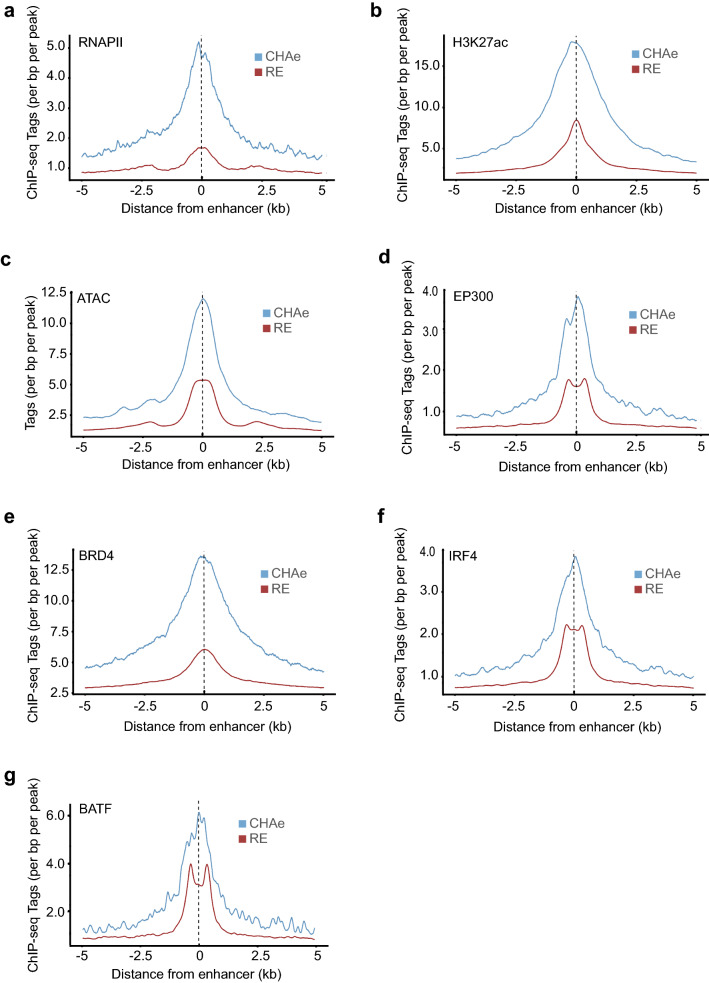
Chromatin and transcription factors in CHA and regular enhancers. Tag density plot centered on CHA and regular enhancers for (**a**) RNA polymerase II occupancy, (**b**) ChIP-seq H3K27ac, (**c**) transposase accessible chromatin, (**d**) EP300 occupancy, (**e**) BRD4 occupancy, (**f**) IRF4 occupancy and (**g**) BATF occupancy. (for all P < 2.2E-16, Wilcoxon rank-sum test). *CHAe* CAGE-tag highly active enhancer, *RE* regular enhancer.

We tested whether the occupancy by the RNA polymerase II (RNAPII) was increased at CHA enhancers. As expected, compared to regular enhancers, CHA enhancers displayed elevated RNAPII ChIP signal (P < 2.2E − 16, Wilcoxon rank-sum test) (Fig. 2a). Signals for H3K27ac and ATAC-seq were also highly increased in CHA vs. regular enhancers (Fig. 2b,c). Analysis of ChIP-seq signal for EP300, a histone 3 acetyl transferase for lysine 27 (H3K27ac), revealed higher occupancy at CHA compared to regular enhancers (P < 2.2E − 16, Wilcoxon rank-sum test) (Fig. 2d). Also, the ChIP-seq tag density for BRD4, a reader of histone acetylation and transcriptional cofactor, was much higher in CHA vs. regular enhancers (P < 2.2E − 16, Wilcoxon rank-sum test) (Fig. 2e). A transcription factor motif analysis showed that several transcription factor motifs such as NF-kappa B, the interferon response factor family, JUN and BATF were enriched in CHA enhancers (Supplementary Table 1). Considering those results, we analyzed ChIP-seq for the transcription factor IRF4 and BATF. This analysis showed significantly elevated ChIP-seq signal for IRF4 and BATF in CHA vs. regular enhancers (Fig. 2f,g). Taken together, these findings highlight a higher recruitment of cell-specific transcription factors at CHA enhancers.

### CHA enhancers are enriched in 3D genomic interactions

**Figure 3:**
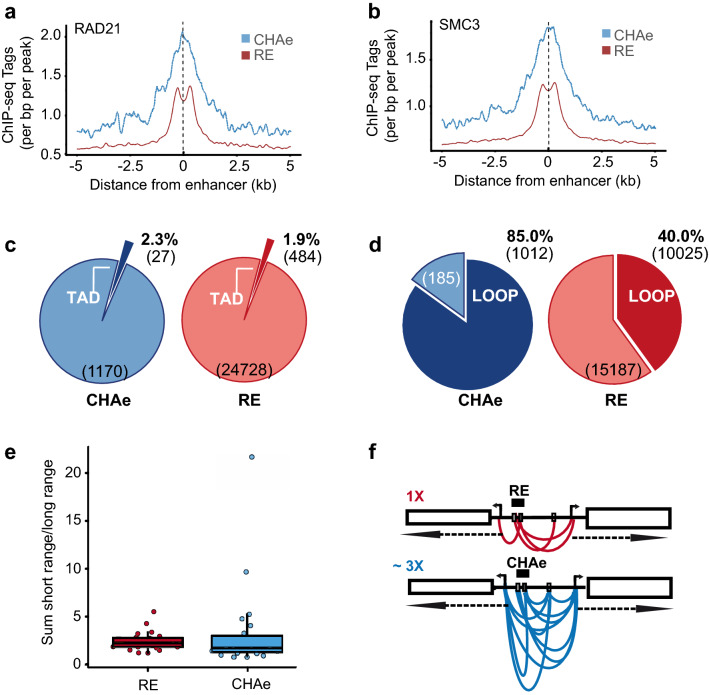
Connectome of CHA and regular enhancers. Tag density plot centered on CHA and regular enhancers for (**a**) RAD21 occupancy (P < 2.2E-16, Wilcoxon rank-sum test) and (**b**) SMC3 occupancy (P < 2.2E-16, Wilcoxon rank-sum test). (**c**) Proportion of enhancers overlapping with TAD (P=0.40, Fisher’s exact test). (**d**) Proportions of enhancers overlapping with anchor loops in H3K27ac-HiChIP (P=1.87E-214, Fisher’s exact test). (**e**) Box-plot representing ratio of the sum of short (<200kb) to long distance (≥200kb) interactions for each chromosome (P=0.38, Wilcoxon rank-sum test). (**f**) Schematic representation of loop density analysis. *CHAe* CAGE-tag highly active enhancer, *RE* regular enhancer, *TAD* topologically associated domain.

Considering that activity of enhancers could be linked to the looping process, we assessed the occupancy of proteins involved in 3D genome organization at CHA and regular enhancers. We documented the ChIP-signal for RAD21 and SMC3, which are members of the cohesin complex. The tag density signals for RAD21 and SMC3 were markedly elevated in CHA compared to regular enhancers (P < 2.2E − 16, Wilcoxon rank-sum test) (Fig. 3a and b). These data thus suggested that CHA enhancers were associated with 3D genome organization. Enhancer-promoter interactions in H3K27ac-HiChIP were analyzed in order to establish genome-wide chromatin interactions of CHA and regular enhancers with the promoter of genes (GSE101498). Analysis of H3K27ac-HiChIP showed that 93% of 1D data overlapped with H3K27ac ChIP-seq data, indicating a good agreement to detect and enrich for active chromatin regions in 3D chromatin conformation assay. By using H3K27ac-HiChIP we identified TADs. The overlapping of enhancers with borders of TADs including a buffer of 10kb was similar for CHA (2.3%) vs. regular (1.9%) enhancers (P=0.40, Fisher’s exact test) (Fig. 3c). These data suggested that CHA enhancers were occupied by essential factors involved in higher order chromatin organization. However, CHA enhancers were not overrepresented at TAD borders. Hence, it is possible that CHA enhancers are not involved in the regulation of long-range looping at domain boundaries, but instead could be involved in enhancer-promoter contact within these domains.

We next analyzed significant loops and their relationships with CHA and regular enhancers. With a stringent loop calling (q-value < 1E-06), we detected 52,175 significant loops. We determined the loops involving CHA and regular enhancers. The proportion of regulatory elements involved in at least one anchor loop was increased for CHA (85%, 1012/1197) vs. regular enhancers (40%, 10025/25212) (P=1.87E − 214, Fisher’s exact test) (Fig. 3d). The short (<200kb) vs. long distance chromatin interaction ratio calculated for each chromosome (“Methods”) involving CHA and regular enhancers were similar (2.4 vs. 3.3, respectively for regular and CHA enhancers, P=0.38, Wilcoxon rank-sum test) (Fig. 3e). However, the mean number of loops per distant regulatory element was markedly increased in CHA versus regular enhancers (12.3±12.7 vs. 4.5±5.1, P<2.2E − 16, Wilcoxon rank-sum test). A schematic representation of anchor loops in a typical CHA enhancer vs. a regular enhancer is depicted in Fig. 3f.

### CHA enhancers in different cell populations are associated with cell-specific functions and open chromatin

In order to assess whether the findings were generalizable, we also mapped CHA enhancers in human coronary artery smooth muscle cells (HCASMC) and normal human epidermal keratinocytes (NHEK). In HCASMC, we mapped 358 CHA enhancers which corresponds to 1.8% of H3K27ac-defined enhancers, whereas in NHEK we identified 852 CHA enhancers (2.5% of enhancers). We found a poor overlap between CHA enhancers and SEs in HCASMC (Jaccard = 0.003) and NHEK (Jaccard = 0.01). Compared to regular enhancers and SEs (n = 1168), the annotation of regulatory regions showed lower P values in CHA enhancers for HCASMC-specific functions such as muscle contraction, regulation of vasoconstriction and regulation of vascular smooth muscle cell proliferation (Suppl. Fig. 3A). Compared to regular enhancers, CHA enhancers in HCASMC were enriched in open chromatin (Suppl. Fig. 3B). Also, occupancy by transcription factors such as JUN and TCF21, which are involved in the regulation of HCASMC function, was markedly increased in CHA versus regular enhancers (Suppl. Fig. 3C,D). Analysis of H3K27ac-HiChIP in HCASMC, showed a higher number of high-confidence anchor loops at CHA compared to regular enhancers (7.7±8.1 loops per CHA enhancer vs. 4.5±5.6 loops per regular enhancer, P<2.2E − 16, Wilcoxon rank-sum test). Among the genes connected to CHA enhancers in HCASMC there are some key transcription factors such as *KLF3*, *SOX15* and *FOXK1*, which are involved myogenic cell fate^23,24^. Consistently, several myogenic genes such as *MYH9*, *MYL12A* and *MYO1C* are also connected to CHA enhancers. The 852 CHA enhancers in NHEK were annotated and enriched for cell-specific function such as skin development, cornification and regulation of hair follicle development in comparison to regular enhancers and SEs (n = 809) (Suppl. Fig. 4A). Compared to regular enhancers, CHA enhancers from NHEK had increased open chromatin and occupancy for MYC, a transcription factor highly expressed in basal cell layer of epidermis and hair follicles (Suppl. Fig. 4B,C)^25^. Also, the occupancy of GRHL3, a transcription factor involved in the differentiation of keratinocyte^26^, was increased in CHA compared to regular enhancers (Suppl. Fig. 4D). Analysis of H3K27ac-HiChIP in HaCaT, a keratinocyte cell line, showed after loop calling a higher number of anchor loops involving CHA compared to regular enhancers (10.1±9.1 loops per CHA enhancer vs. 6.1±6.0 loops per regular enhancer, P<2.2E − 16, Wilcoxon rank-sum test). In HaCaT, gene promoters connected to CHA enhancers include several genes involved in the cornification process such as genes located in the human keratin cluster locus at 12q13 (*KRT80*, *KRT7*, *KRT83*, *KRT84*, *KRT5*, *KRT72* and *KRT8*) as well as keratin-associated proteins genes (*KRTAP*) (Suppl. Fig. 5)^27^. The Jaccard index for the CHA enhancers between GM12878 versus HCASMC (0.016), GM12878 versus NHEK (0.018) and HCASMC versus NHEK (0.069) showed that CHA enhancers between these three cell types were largely unique and cell type specific (Suppl. Fig. 6). Taken together, these data highlight that CHA enhancers are related to cell identity and have a high occupancy of transcription factors and are highly connected to gene promoters with cell-specific functions.

### Hierarchy among the connectome of CHA enhancer-promoter

**Figure 4:**
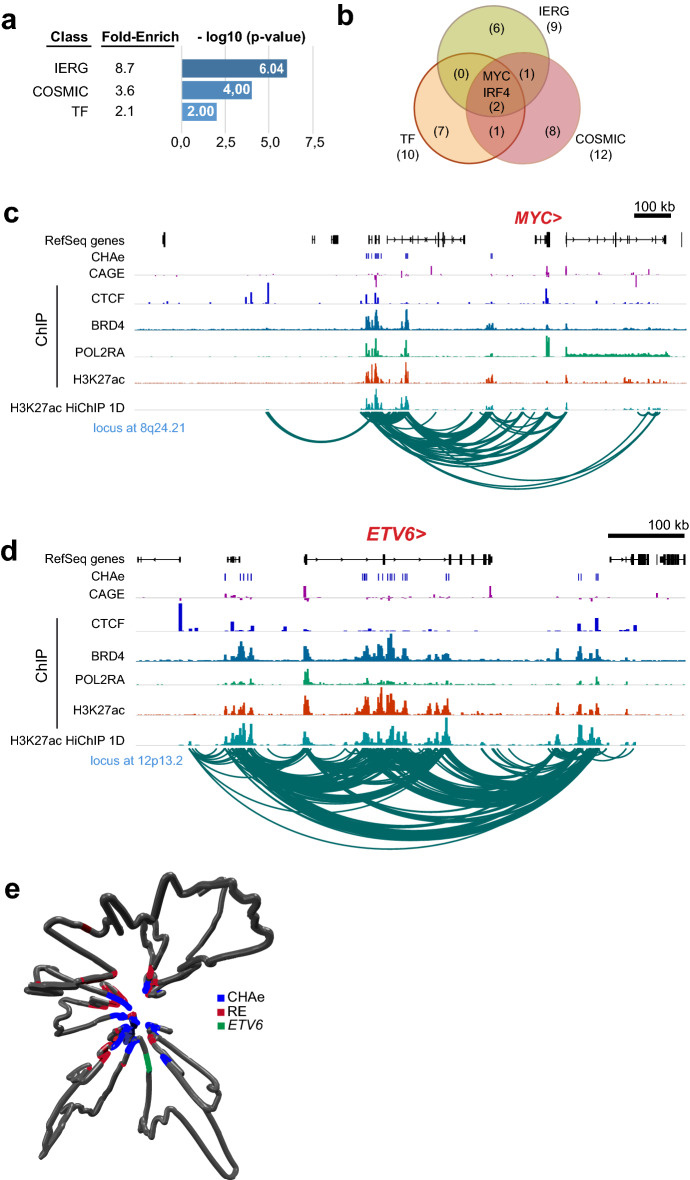
Characterization of hub and non-hub CHA regulatory units. (**a**) Enrichment of genes mapped by hub CHA regulatory unit. (**b**) Representation in Venn diagram of transcription factors (TF), genes involved in cancer (COSMIC) and IERG mapped by hub CHA regulatory unit. (**c,d**) Representative loci of hub CHA regulatory units; tracks represent genes, CHAe, CAGE, ChIP-seq CTCF, ChIP-seq BRD4, ChIP-seq POL2RA, ChIP-seq H3K27ac, H3K27ac-HiChIP 1D and arcs of significant loops; mapped genes are in red (Integrative Genomics Viewer). (**e**) 3D DNA modelling structure of hub CHA regulatory unit for *ETV6*; green: promoter region of *ETV6*; blue: CHAe; red: RE. *CHAe* CAGE-tag highly active enhancer, *RE* regular enhancer, *IERG* immediate early response gene, *TF* transcription factor.

Enhancers interacting with gene promoters, which are part of the cell chromatin connectome, form dense networks of regulatory units. According to the level of connection, distant regulatory elements and their target gene promoters form hierarchical networks with functional relevance^15^. We hypothesized that among the gene promoters connected to CHA enhancers those with the highest level of contact with CHA elements formed regulatory hubs, which may include genes of significant relevance for cellular functions. We defined hub CHA regulatory units as enhancers and promoters with the highest number of CHA loops converging on promoters (≥ 90th percentile), which were compared to non-hub CHA regulatory units (CHA enhancer-promoter loops <90th percentile). In total, hub CHA regulatory units mapped 89 unique highly connected gene promoters. Genes within hub CHA regulatory units were enriched in immediate early response genes (fold-enrichment: 8.7, P=9.07E − 07, hypergeometric test), in genes involved in cancer (COSMIC database) (fold-enrichment: 3.6, P=0.0001, hypergeometric test) and encoding for transcription factors as reported in the TF checkpoint database (fold-enrichment: 2.1, P=0.01, hypergeometric test) (Fig. 4a). Among the different genes within hub CHA regulatory units, *MYC* and *IRF4* are unique as they are transcription factors, immediate early response genes and are involved in cancer (Fig. 4b). Figure 4c shows the locus at 8q24.21 where the promoter of *MYC* has many loops with highly interconnected CHA enhancers (CHA enhancers at ~550–160kb from the promoter). One of the most connected protein coding gene in hub CHA regulatory units is *ETV6*, which encodes for a transcriptional repressor involved in hematopoiesis and leukemia^28^. Figure 4d represents the locus at 12p13.2 where *ETV6* is densely connected to distant regulatory elements. Dynamic modelling of the enhancer-promoter 3D structure showed a multi loop aggregate forming a rosette-like structure at hub CHA regulatory unit for *ETV6*, which spans more than 500kb (Fig. 4e).

### Hub CHA regulatory units and transcription elongation

**Figure 5:**
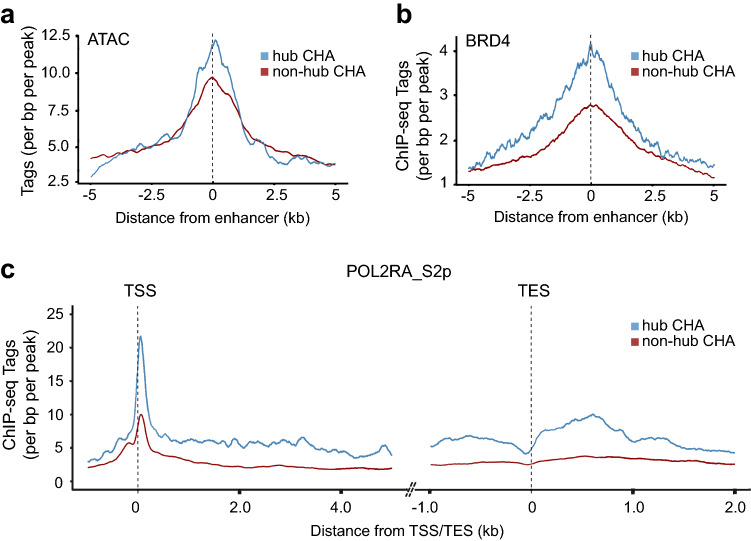
Chromatin landscape and recruitment of RNA polymerase at hub and non-hub CHA regulatory units. (**a**) Tag density plot of ATAC-seq in hub CHA versus non-hub CHA (P=0.001, Wilcoxon rank-sum test). (**b**) Tag density plot of ChIP-seq BRD4 in hub and non-hub CHA regulatory unit (P < 2.2E-16, Wilcoxon rank-sum test). (**c**) Metagene analysis of RNAPII phosphorylated on serine 2 (POL2RA_S2p) in hub versus non-hub CHA regulatory unit.* TSS* transcription start site,* TES* transcription end sites (P < 2.2E − 16, Wilcoxon rank-sum test).

We next hypothesized that hub CHA regulatory units include elements with increased active chromatin features. Among CHA enhancers, hub regulatory elements had increased open chromatin and occupancy by BRD4 (Fig. 5a,b). Considering the previous finding, we wondered whether gene promoters within hub CHA regulatory units may exhibit a higher recruitment of molecules involved in the transcription process. We thus assessed the recruitment of RNAPII phosphorylated on serine 2 (RNAPII-S2p), which is a marker of the elongation phase during the transcription process, at genes within hub CHA regulatory units^29^. A metagene analysis revealed that the recruitment of RNAPII-S2p was increased at the transcription start site (TSS) and gene body of genes within hub CHA regulatory units vs. non-hub CHA regulatory units (Fig. 5c). To further assess if genes within hub CHA regulatory units were associated with increased processivity of the RNAPII, we analyzed global run-on sequencing (GRO-seq) data (GSE60454). Genes with nonzero tag density at the TSS (− 50bp and +300bp) were considered for the analysis (“Methods”). We calculated the pause index, which is the ratio of RNAPII tag signal at the TSS to the gene body. The pause index was significantly increased at the promoter of genes connected to non-hub CHA vs. hub CHA regulatory units (median [95% CI]: 5.15 [4.61–5.78] vs. 2.96 [2.22–4.43], P=0.02, Wilcoxon rank-sum test). When using a cut-off of 2 to assess paused genes (pause index > 2)^30^, there was a trend for a higher proportion of paused genes in non-hub CHA vs. hub CHA regulatory units (73% vs. 64%, P = 0.096, Fisher’s exact test). These data suggested that genes within hub CHA regulatory units are less likely to be paused.

### CHA enhancers and autoimmune disorders

**Figure 6:**
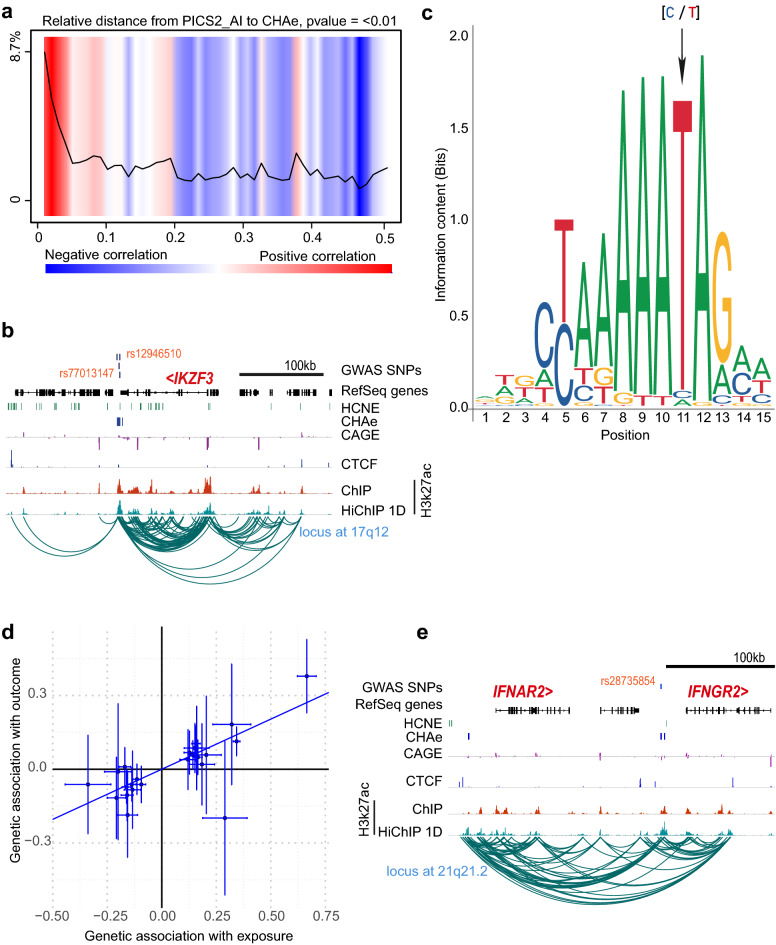
Relationships between CHA enhancers and gene variants for autoimmune diseases. (**a**) Heat map representing the relative distance between Probabilistic Identification of Causal SNPs (PICS) autoimmune variants and CHA enhancers (P < 2.2E-16, Kolmogorov-Smirnov test) (GenometriCorr). (**b)** *IKZF3* risk locus for lupus erythematosus; tracks represent SNPs, genes, HCNE, CHA enhancers, CAGE, ChIP-seq CTCF, ChIP-seq H3K27ac, H3K27ac-HiChIP 1D and arcs of significant loops; mapped genes are in red. (**c**) Sequence logo for MEF2C; arrow indicates rs12946510 base position where the reference allele is C and the risk allele is T. (**d**) Inverse variance weighted MR for *IKZF3* in systemic lupus erythematosus. (**e**) *IFNAR2/IFNGR2* risk locus for rheumatoid arthritis; tracks represent SNPs, genes, HCNE, CHA enhancers, CAGE, ChIP-seq CTCF, ChIP-seq H3K27ac, H3K27ac-HiChIP 1D and arcs of significant loops; mapped genes are in red. *CHAe* CAGE-tag highly active enhancer, *HCNE* human conserved noncoding elements.

Gene variants associated with complex trait disorders are enriched in the noncoding genome. We wondered whether autoimmune gene variants were enriched in CHA enhancers. Autoimmune variants prioritized from the Probabilistic Identification of Causal SNPs (PICS) were downloaded for the analysis (“Methods”)^31,32^. We found that PICS autoimmune variants resided at proximity (P<2.2E − 16, relative distance, Kolmogorov-Smirnov test) and within CHA enhancers (fold-enrichment:10.4, P<2.2E − 16, binomial test) (Fig. 6a) (“Methods”). By using CHA enhancer-promoter looping, we mapped 138 autoimmune variants to gene promoters (Supplementary Table 2). For several variants, genes mapped by chromatin looping were upstream or downstream from the closest annotated gene to the SNP (Supplementary Table 2). We next ask whether genes mapped by CHA enhancer-promoter contacts may be causal in Mendelian Randomization. For each gene-disease pair mapped by CHA enhancer-promoter looping, we performed Mendelian randomization analysis for 4 major autoimmune disorders (rheumatoid arthritis, systemic lupus erythematosus, inflammatory bowel disease, type 1 diabetes) (“Methods”). Blood expression quantitative trait loci (eQTL) from eQTLGen, the largest blood eQTL resource including 31,684 samples, was leveraged to assess the exposition. In total, Mendelian randomization could be performed (independent instrumental variables ≥ 3, P_eQTL_ for instrumental variables <5E-08) for 40 CHA enhancer 3D mapped gene-disease pairs. We found that 33% (13/40) of CHA enhancer 3D mapped genes were significant causal candidates for 4 major autoimmune disorders (P_causal_<0.05) (Supplementary Table 3). For instance, PICS variants rs77013147 and rs12946510 at 17q12 are associated with several autoimmune disorders. Gene variants rs77013147 and rs12946510 are within CHA enhancers, which have chromatin loops with the promoter of *IKZF3* (Fig. 6b). The alleles T-rs12946510 (P_eQTL=_1.42E − 243) and C-rs77013147 (P_eQTL=_4.71E − 190) are associated with a higher blood expression of *IKZF3*. Motif analysis showed that the gene variant rs12946510 is within a transcription factor motif in which the risk allele T (freq EUR=0.47) (Supplementary Fig. 7) increases the binding for MEF2C (Fig. 6c), a transcription factor involved in B cell proliferation^33^. This region is localized in a highly conserved noncoding region between human and mouse (Fig. 6b). *IKZF3* encodes for an IKAROS family zinc finger 3 protein, which is involved in lymphocyte proliferation^34^. In Mendelian randomization, the blood expression of *IKZF3* was positively associated with the risk of systemic lupus erythematosus (beta=0.41, SE=0.05, P_IVW_= 1.01E − 16) (Fig. 6d). At 21q21.2, PICS variant A-rs28735854 (freq EUR=0.15) is associated with rheumatoid arthritis. At this risk locus, intergenic variant rs28735854 is within a CHA enhancer having loops with the promoters of *IFNGR2* and *IFNAR2* (Fig. 6e). In Mendelian randomization, the expression of *IFNGR2* (beta=− 0.12, SE=0.02, P_IVW_= 7.78E − 12) and *IFNAR2* (beta=− 0.08, SE=0.03, P_IVW_=0.004) in blood cells were negatively associated with the risk of rheumatoid arthritis.

## Discussion

In the present work, we underlined that enhancers with a high level of transcriptional activity as determined by CAGE, referred to as CHA enhancers, were associated with active chromatin features and the recruitment of cell-specific transcription factors. We underlined novel findings where CHA enhancers were cell-specific and did not significantly overlap with SEs. Compared to regular enhancers and SEs, CHA enhancers had lower GO P-values for cell identity functions. Hub CHA regulatory units established a dense network of enhancer-promoter interactions and were connected to immediate early response genes and transcription factors. Genes within hub CHA regulatory units had higher transcription elongation. CHA enhancers were enriched in autoimmune-associated gene variants prioritized by PICS and enhancer-promoter mapping identified causal candidate genes. Overall, these data underlined that highly transcribed enhancers establish a dense connectome with regulatory activity and having implications on cellular functions and disorders.

We observed that CHA enhancers were conserved and enriched in CGI. These findings suggested that CHA enhancers may exert a control over key regulatory genes. To this effect, we found lower P-values in cell-specific GO for CHA vs. regular enhancers and SEs. CGI are enriched at gene promoters and have been shown to support transcriptional activity^35^. Though regular enhancers were largely poor in CGI, CHA enhancers were significantly enriched in CGI. A recent analysis has underlined that a small subset of enhancers characterized by the presence of CGI were stronger, enriched in transcription factor binding and highly conserved^36^. These data are in line with the present findings and suggest that CHA enhancers define a subset of active regulatory elements likely involved in cell-specific functions and development.

Previous work conducted in GM12878 underlined that transcriptionally active regulatory elements are enriched in the recruitment of transcription factors^37^. Herein, we provide the confirmation in GM12878 that CHA enhancers are associated with higher recruitment of cell-specific transcription factors and we expand these findings to HCASMC and epidermal keratinocytes as a proof of generalization. These data support that a high level of transcription in H3K27ac defined enhancers is associated with chromatin remodelling in genomic regions enriched for cell-specific functions. A growing body of evidence suggest that enhancer-promoter interactions, which control gene expression, is actively regulated by the recruitment of several molecules including 3D architectural proteins^4,38^. Data also suggests that transcriptional activity and noncoding RNAs participate to the establishment of genomic interactions^39^. The present work supports that a high level of transcription in enhancers is associated with a higher density of genomic interactions. CHA enhancers were associated with an elevated recruitment of 3D architectural molecules (RAD21 and SMC3) and with higher density of genomic loops.

Among the CHA enhancers, we found that densely connected elements and forming hub CHA regulatory units were connected to gene promoters of immediate early response genes and transcription factors. Some genes within hub CHA regulatory units, such as *ETV6,* were connected with several regulatory elements and formed a rosette-like structure spanning several hundred of kilobases. These data suggested that some genes with important functions have connections with multiple highly transcribed enhancers. Multiple enhancers converging on gene promoters may afford robustness with lesser impact of stochastic events on the control of gene expression^40^. Among the gene promoters within hub CHA regulatory units, several were early immediate response genes with roles in the inflammatory response. We found that hub CHA enhancers had increased open chromatin and recruitment of BRD4, a transcriptional cofactor^41^. Of significance, genes within hub CHA regulatory units had higher recruitment of elongating RNAPII. Moreover, these genes had lower pause index. Hence, hub regulatory units including highly transcribed enhancers is associated with an increased transcription elongation at target genes necessitating robust gene control.

The majority of gene variants associated with complex trait disorders are located in the noncoding genome^42^. Studies have consistently shown that noncoding gene variants are enriched in regulatory elements^43^. Herein, we found a strong enrichment of PICS autoimmune variants in CHA enhancers. Enhancer-promoter mapping of autoimmune SNPs showed that CHA enhancers were often connected to genes located several kilobases upstream or downstream from the closest annotated gene, which is an accordance with previous studies. Hence, 3D mapping of CHA enhancers may help identify causal candidate genes in genome-wide association studies and reposition drugs for novel treatment opportunities. Consistently, a significant proportion of disease-associated variants mapped by CHA enhancer-promoter contacts identified causal candidate genes in Mendelian randomization. For instance, *IKZF3*, which was mapped by CHA enhancers, is a strong causal candidate for systemic lupus erythematosus as shown by Mendelian randomization. Lenalidomide, which is approved in the treatment of multiple myeloma, targets IKZF3 for degradation^44^. Drugs targeting this pathway could thus be repositioned for the treatment of lupus^45^.

The present work provided evidence that a subset of enhancers typified by a very high transcriptional activity are associated with active chromatin features and the recruitment of cell-specific transcription factors. We show that CHA enhancers do not appreciably overlap with SEs and provide better assessment of cell-specific functions. Hierarchy among the CHA enhancers was observed as hub CHA regulatory units were associated with higher transcription elongation at connected genes. CHA enhancers may help establish 3D genome regulatory networks involved in the control of gene expression during development and under pathologic conditions.

## Methods

### CAGE

CAGE normalized expression matrix for the human enhancers was downloaded from the Functional Annotation of the Mammalian Genome (FANTOM5) and data for GM12878 were extracted. Replicates (n=3) were combined by calculating the mean for CAGE-tag value at each annotation with at least one nonzero value. CAGE bam hg19 files were downloaded from FANTOM5. Bam replicates were sorted and merged before being converted to sam with samtools (v1.10). These files were processed with HOMER (v4.11) using makeUCSCfile for each strand and combined into a bedgraph for data visualization in Integrative Genomics Viewer (IGV)^46^.

### Processing of ChIP-seq and ATAC-seq

Replicate FASTQ files from GM12878 for ATAC-seq (ENCSR095QNB) and ChiP-seq for H3K27ac (ENCSR000AKC), POLR2A/RNPAII (ENCSR000DKT), POLR2A_phosphoS2 (ENCSR000DZK), EP300 (ENCSR000DZD), IRF4 (ENCSR000BGY), BATF (ENCSR000BGT), SMC3 (ENCSR000DZP0), RAD21 (ENCSR000EAC) and CTCF (ENCSR000DZN) were downloaded from the Encyclopedia of DNA Elements (ENCODE). SRA files from ChIP-seq for BRD4 were downloaded from GEO DataSets (GSE62912). For HCASMC, SRA files from GSE1369 (ChIP-seq JUN), GSE124011 (ChIP-seq TCF21 and H3K27ac, ATAC-seq) were downloaded. For NHEK, FASTQ files from ENCODE (ENCSR000ALK) (ChIP-seq H3K27ac), (ENCSR000DCY) (FAIRE-seq) and SRA files from GSE32883 (ChIP-seq MYC) and GSE94471 (ChIP-seq GRHL3) were downloaded. Parallel-fastq-dump (v0.6.5) was used to extract FASTQ from SRA files. FASTQ files were aligned with Bowtie2 (v2.3.5.1) on hg19 and converted to bam files with samtools. Duplicate reads were removed with picard-tools and replicates were merged. The bigWig tracks were generated with deepTools (v3.3.2) bamCoverage and the sorted bam files^47^; bigWig files were visualized in IGV along with h19 gene annotations. Peak call for H3K27ac ChIP was performed with MACS2 (v2.2.7.1) with a threshold q-value at 1E-05 and with a normalization for the input. H3K27ac peaks overlapping the promoter of protein coding genes (TSS±2kb) (GENCODE version 35 in build 37) and the ENCODE blacklist regions were removed by using bedtools (v2.27.1) to obtain the H3K27ac defined enhancers.

### Super-enhancers, gene ontology and motifs analyses

Super-enhancers (SEs) annotation was performed by using HOMER findPeaks.pl with the option -style super, data was normalized by input (− i). Jaccard index between annotations were performed by using bedtools with the jaccard option. Gene ontology for annotations was performed by using HOMER annotatePeaks.pl function with the option -go. Transcription factor motif enrichment analysis for annotations was performed by using HOMER findMotifsGenome.pl^48^.

### CGI and human conserved noncoding regions

Bed files for CGI and human conserved noncoding elements between man and mouse (HCNE) were downloaded from UCSC and the Atlas of Noncoding Conserved Regions in Animals (ANCORA) respectively^49^. The percentage of referent annotations (CHA and regular enhancers) overlapping with CGI and HCNE (queries) was determined by using the midpoint of query. If a referent annotation had more than one overlap with a query it was reported only once (bedtools intersect with -u option). Proportions of overlap between each group of referents (CHA vs. regular enhancers) were compared with the Fisher’s exact test performed with the scipy library in Python (3.8.10).

### Tag density plot and heat map

Aligned sam files were normalized for a total of 10 million tag counts and processed with HOMER^48^ makeTagDirectory function with unique reads and MAPQ score > 10. Tag density values centered on annotations were generated by using HOMER with the annotatePeaks.pl function using bins of 10 (-hist 10) and covering 10 kb (-size 10000). Tag count normalized matrix of ChiP-seq processed by HOMER were subtracted by the tag count matrix of input. Plots were generated with ggplot2 in R (4.0.3). Heat map centered on annotations was performed by using deepTools; deepTools computeMatrix function was performed with the ATAC-seq bigWig file and the heat map was generated with the plotHeatMap function. Tag values obtained from HOMER between different annotations were compared by a Wilcoxon rank-sum test performed in R.

### Analysis of HiChIP

FASTQ files from H3K27ac-HiChIP for GM12878, HCASMC (GSE101498) and HaCaT (GSE151193) were downloaded and aligned by using HiC-Pro (2.11.4) with default settings^50^. The 1D track for H3K27ac was generated by using deepTools bamCoverage from sorted bam files obtained from HiC-Pro. Loop call was performed with FitHiChIP (v10.0) with default settings and high confidence loops (q-value < 1E − 06) were identified for downstream analyses. Identification of loops overlapping with regulatory elements and gene promoters was performed by using bedtools with the intersect function. Gene promoters were annotated by using a region ± 2kb from the transcription start site and GENCODE version 35 in build 37. TADs were determined by using HiCExplorer (v2.2.1.1) hicFindTADs function and adding a buffer of 10kb. For each chromosome, the ratio of the sum of short (< 200 kb) to long distance (≥ 200 kb) interactions were calculated for loops involving CHA and regular enhancers.

### Modelling of 3D interactions

The modelling of 3D interactions was performed by using CSynth^51^. CSynth provides an interactive interface to perform in real-time the modelling of chromatin fiber interactions derived from HiC technology. Annotation file in bed format of the region of interest (ROI) was created to which RGB color code was associated for each functional element (enhancers and gene promoters). A contact file of the ROI was obtained from the normalized interaction file from FitHiChIP. CSynth was used with default value parameters except for pushapartforce (set at 75).

### Enrichment tests

Immediate early response genes curated by Arner E. et al. were retrieved for enrichment analysis^12^. Genes associated with cancer from the COSMIC database^52^ and transcription factors curated by TFcheckpoint were downloaded^53^. Genes to be tested were intersected with annotated gene lists and hypergeometric tests were performed by using the Python library scipy.stats. Whole genome protein coding genes were used as the background.

### Metagene analysis

Transcription start (TSS) and end sites (TES) of mapped genes on each strand were processed by HOMER annotatePeaks.pl (-hist 10) using the POL2RA_phosphoS2 normalized data obtained with HOMER makeTagDirectory with unique reads and MAPQ score >10. Data were combined into a metagene tag file around the TSS (− 1000 and +5000) and TES (− 1000 and +2000). Plots were generated with ggplot2 in R. Tag values between different annotations were compared by a Wilcoxon rank-sum test performed in R.

### GRO-seq

SRA files from GRO-seq data in GM12878 (GSE60454) were downloaded and converted to FASTQ files with parallel-fastq-dump. Files were trimmed with cutadapt and aligned with Bowtie2 on hg19. Pause index was calculated as the ratio of reads centered on the TSS including a window of (− 50bp to +300bp) to the gene body normalized for its length^54^. Files in sam format were normalized and processed with HOMER with makeTagDirectory and analyzeRNA.pl -pausing 300. Genes having nonzero tag values at the promoter were retrieved to obtain the pause index from the HOMER output file and compared between groups with the Wilcoxon rank-sum test performed in R. Genes in pause were identified by using a pause index > 2 and compared between groups with a Fisher’s exact test performed with the Python library scipy.stats.

### Analysis with autoimmune gene variants

GWAS autoimmune variants processed by the Probabilistic Identification of Causal SNPs (PICS)^32^ were downloaded. PICS is a Bayesian approach for the fine-mapping of causal candidate variants based on the linkage disequilibrium (LD) and the association data. PICS autoimmune data were processed from the GWAS catalog as of January 11, 2019. Autoimmune diseases with at least 5 significant genome-wide significant variants (P_GWAS_<5E-08) were processed. Variants were clumped with PLINK at r^2^ 0.5 within a 250 kb window and were processed by running PICS2^31^. Variants with PICS probability ≥ 0.025 were kept as candidate causal SNPs. Enrichment of autoimmune PICS variants (query) in annotation (referent) was performed by using the R package GenometriCorr^55^. From the output of GenometriCorr, we reported the relative distance Kolmogorov-Smirnov test and the projection test (binomial test). Variants overlapping with CHA enhancers and connected to gene promoters were assessed for a transcription factor motif analysis with the Predicting Regulatory Functional Effect by Approximate P-value Estimation (PERFECTOS-APE) by using the HOCOMOCO-11 human collection. PERFECTOS-APE is a prediction algorithm to assess regulatory role of SNPs on transcription factor binding sites.

### Mendelian randomization

CHA enhancer-promoter looping mapped genes for 4 major autoimmune disorders (rheumatoid arthritis^56^, systemic lupus erythematosus^57^, inflammatory bowel disease^58^, type 1 diabetes^59^) were considered for causal inference in Mendelian Randomization (data were downloaded from the GWAS Catalog; see URLs). In cases where genes mapped by variants were associated to ‘Autoimmunity (multiple disease)’ in PICS processed data we considered these genes potentially associated with the 4 major autoimmune disorders. Two-sample Mendelian randomization by using multiple *cis* instruments was performed by using blood eQTLs from eQTLGen as the exposition^60^. SNPs associated with the blood expression (P_eQTL_<5E-08) within a window of 500kb from the TSS of the gene of interest and independent (r^2^ < 0.1 based on the 1000G EUR reference panel) were selected as instrumental variables. SNPs were clumped with PLINK1.9 and using the genotypes of European population in the 1000 Genomes Project. Mendelian randomization was performed for genes with at least 3 instrumental variables with the inverse variance weighted (IVW) method obtained from the R package MendelianRandomisation. Gene-disease pairs nominally significant (P_IVW_ < 0.05) in Mendelian randomization were considered as potential causal candidates.

### Ethics approval and consent to participate

Datasets from ENCODE, GEO DataSets, FANTOM5, eQTLGen and PICS were publicly available and did not require ethical approval.

## Supplementary Information

Supplementary Table 1.

Supplementary Table 2.

Supplementary Table 3.

Supplementary Figures.

Supplementary Legends.

## Data Availability

All the data are available in the manuscript including in the supplementary tables and figures. Sequencing files from ENCODE, FANTOM5 and GEO DataSets were publicly available. Accession number and URLs are provided in the manuscript “Methods” section.
