# Supplementary material for: Connectome and regulatory hubs of CAGE highly active enhancers
Source: Sci Rep. 2023 Apr 5;13:5594. doi: 10.1038/s41598-023-32669-3 (PMC10076288; doi:10.1038/s41598-023-32669-3)

Supplementary Figure 1: Correlation of CAGE with H3K27ac in enhancers (GM12878).
(Deeptools).

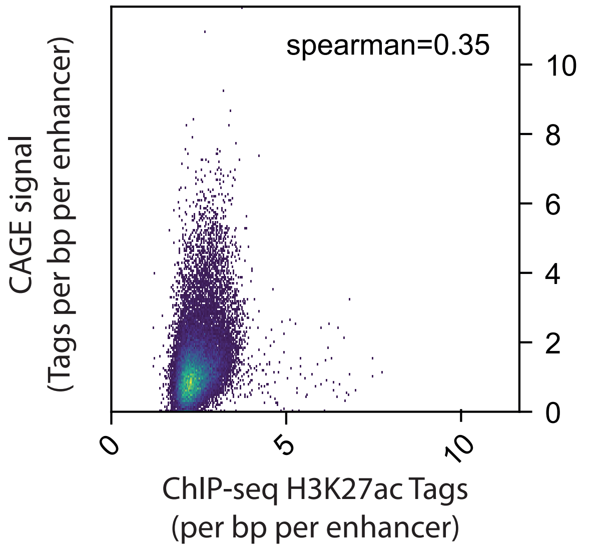

Supplementary Figure 2: Open chromatin in enhancer according to CAGE activity.
Tag density plot centered on enhancers according to CAGE activity for all the enhancers (total), nonzero CAGE activity, CAGE ≥70th percentile and CAGE ≥90th percentile.

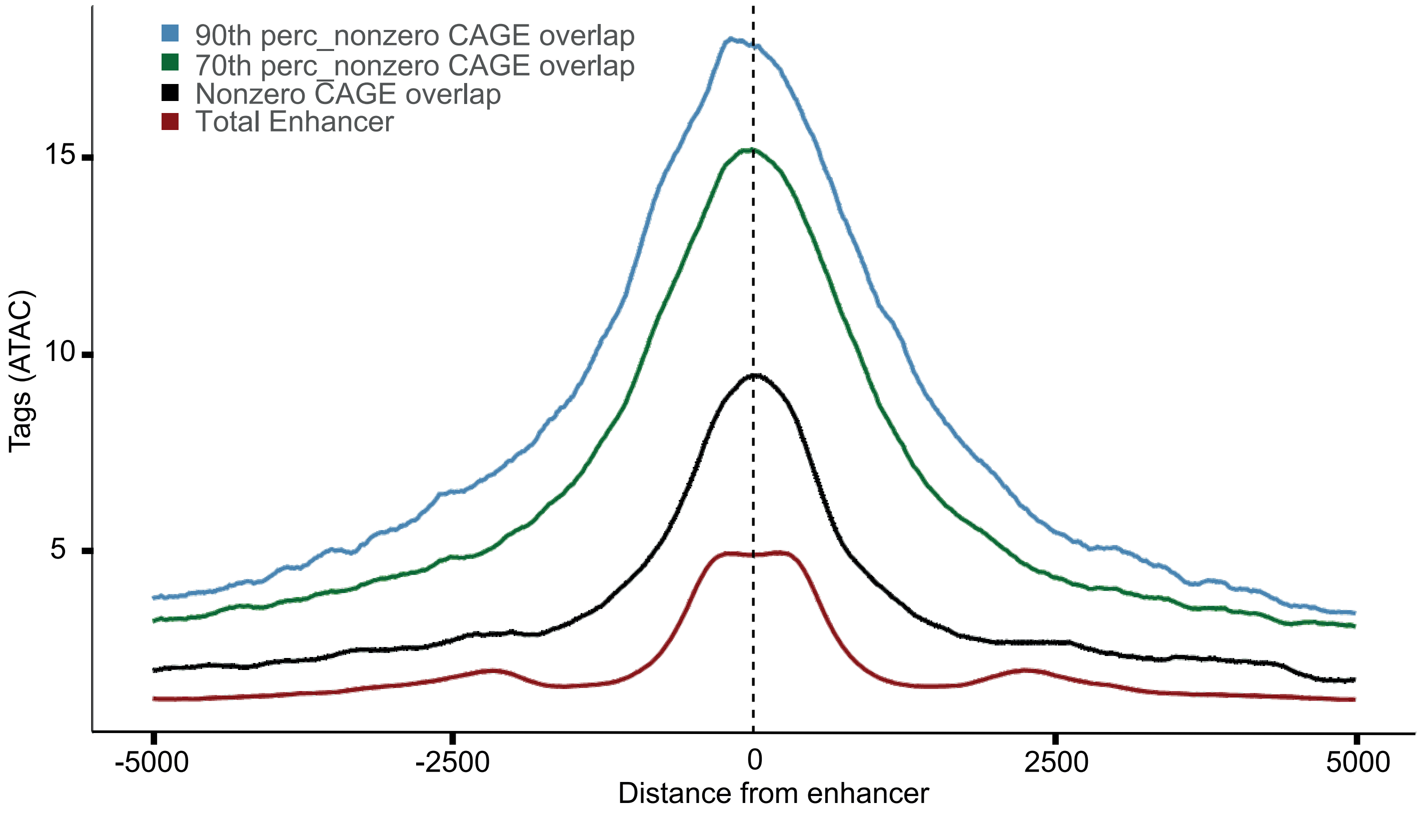

Supplementary Figure 3: CHA versus regular enhancers for HCASMC. A) Enrichment for smooth muscle cell functions in CHA (CHAe), regular enhancers (RE) and super-enhancers (SE). Tag density plot centered on CHA and regular enhancers for **B)** open chromatin (ATAC-seq), **C)** JUN occupancy and **D)** TCF21 occupancy. (for B to D, P < 2.2E-16, Wilcoxon rank-sum test).

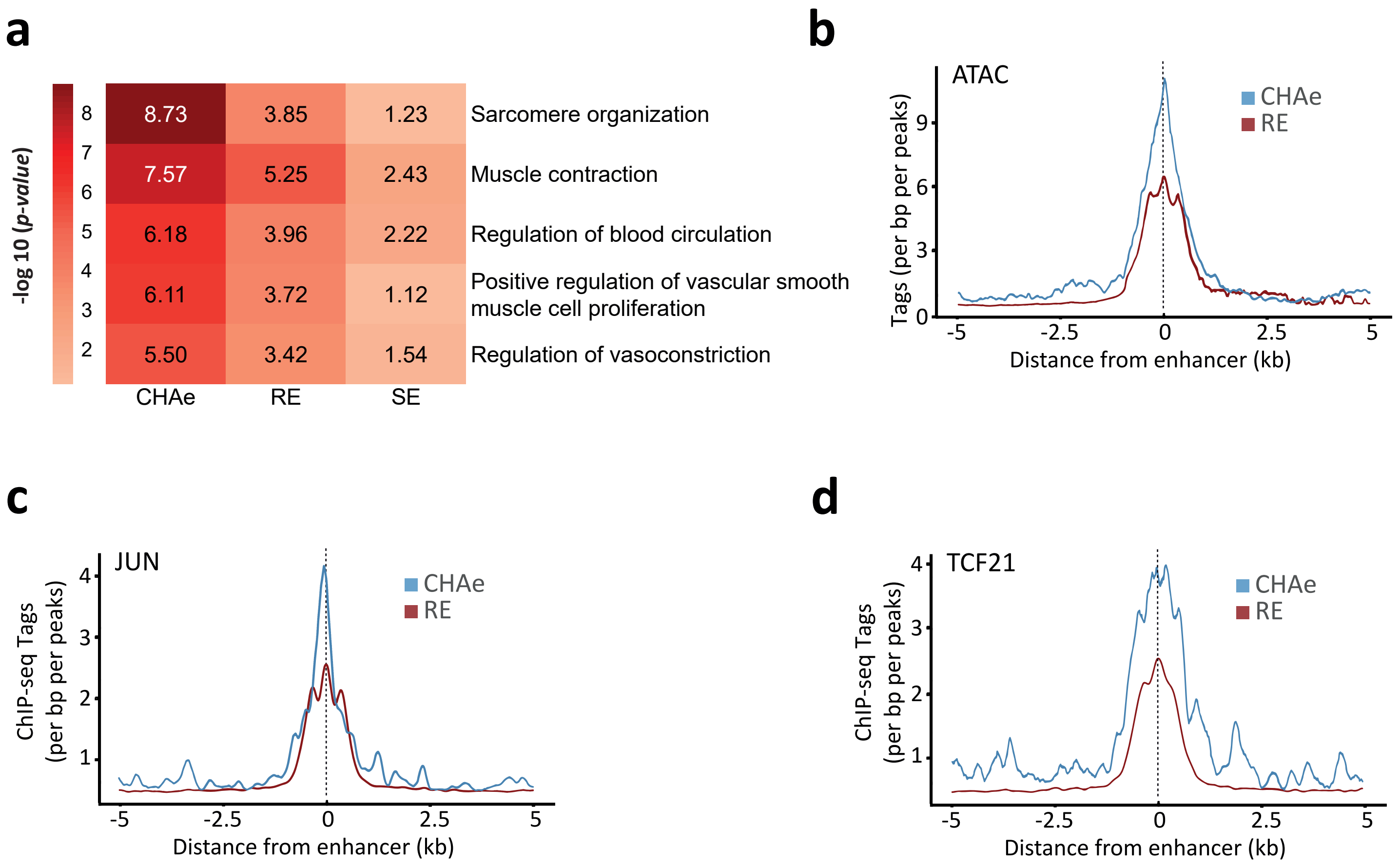

Supplementary Figure 4: CHA versus regular enhancers for NHEK. A) Enrichment for keratinocyte functions in CHA (CHAe), regular enhancers (RE) and super-enhancers (SE). Tag density plot centered on CHA and regular enhancers for **B)** open chromatin (FAIRE-seq), **C)** MYC occupancy and **D)** GRHL3 occupancy. (for B to D, $P < 2.2E-16$, Wilcoxon rank-sum test).

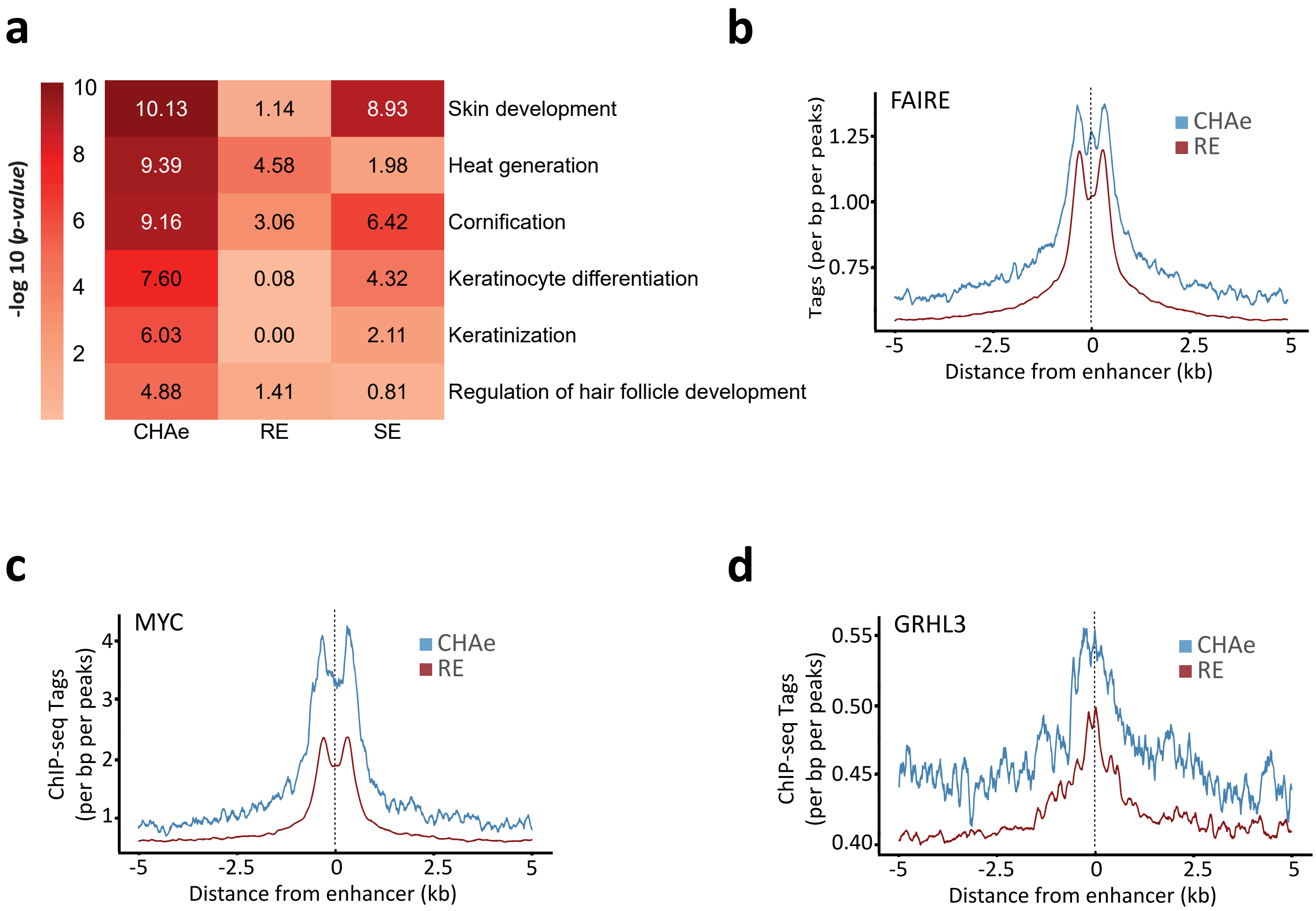

	CHAe	RE	SE
Skin development	10.13	1.14	8.93
Heat generation	9.39	4.58	1.98
Cornification	9.16	3.06	6.42
Keratinocyte differentiation	7.60	0.08	4.32
Keratinization	6.03	0.00	2.11
Regulation of hair follicle development	4.88	1.41	0.81

Supplementary Figure 5: KRT locus in HaCaT. Representation of hub CHA regulatory unit at KRT locus; tracks represent genes, CHAe, CAGE, ChIP-seq CTCF, ChIP-seq MYC, ChIP-seq GRHL3, ChIP-seq POL2RA, ChIP-seq H3K27ac, H3K27ac-HiChIP 1D and arcs of significant loops; mapped genes are in red (IGV).

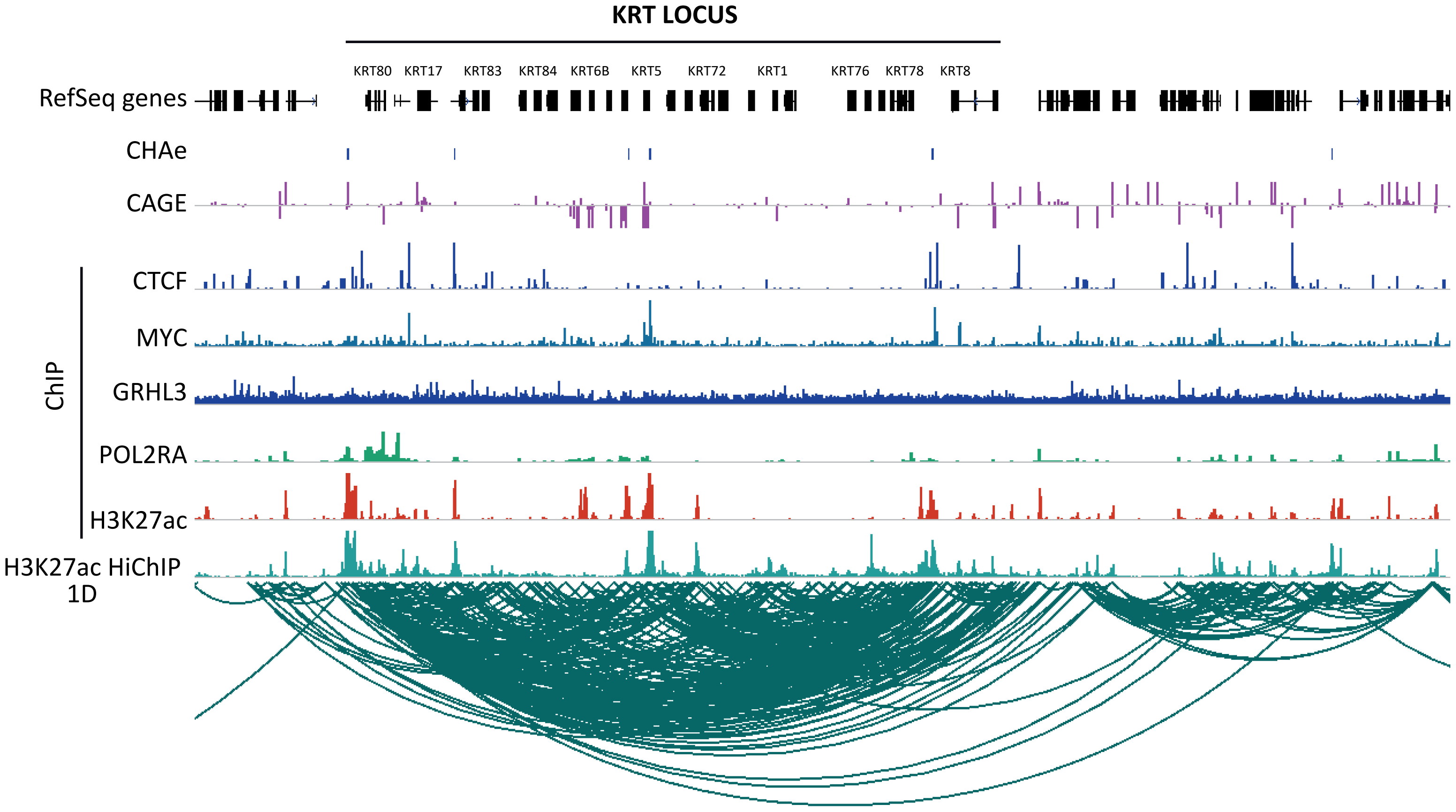

Supplementary Figure 6: Comparison of cell type specificity of CHAe. Tracks represent genes, CHAe, ATAC, ChIP-seq H3K27ac for GM128, HCASMC, NHEK (IGV).

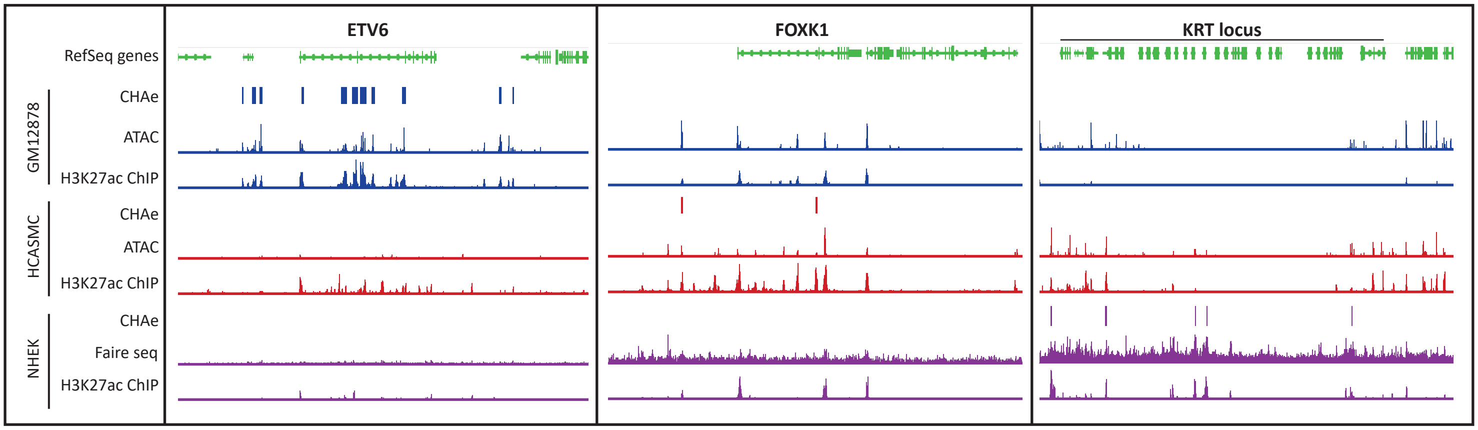

Supplementary Figure 7: Allele frequency for rs12946510 in the 1000 genome. Blue represent the risk allele T-rs12946510 (from Geography of Genetic Variants Browser).

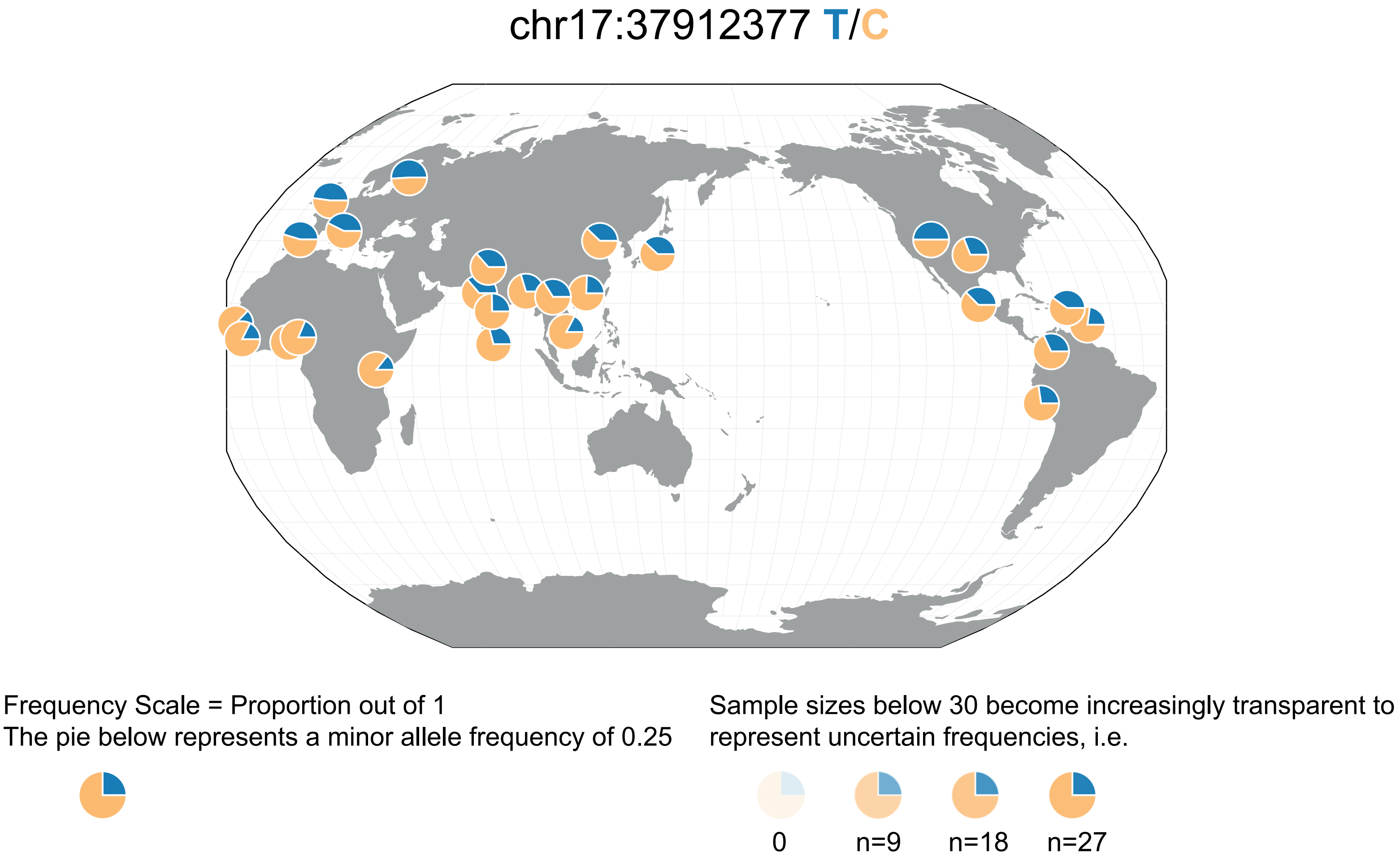

Supplement: Supplementary file 4 — Supplementary Figures. [file 41598_2023_32669_MOESM4_ESM.pdf]
