# Supplementary material for: Connectome and regulatory hubs of CAGE highly active enhancers
Source: Sci Rep. 2023 Apr 5;13:5594. doi: 10.1038/s41598-023-32669-3 (PMC10076288; doi:10.1038/s41598-023-32669-3)
Supplement: Supplementary file 5 — Supplementary Legends. [file 41598_2023_32669_MOESM5_ESM.docx]

**Supplementary table legends**

**Supplementary Table 1:** Transcription factor motif analysis for CHA enhancers.

**Supplementary Table 2:** PICS autoimmune variants mapped by CHA enhancers and chromatin looping.

**Supplementary Table 3:** Mendelian randomization for blood expressed genes (eQTLGen) and CHA enhancer 3D mapped gene-disease pairs.
